# Comorbid disease burden among MS patients 1968–2012: A Swedish register–based cohort study

**DOI:** 10.1177/1352458520910497

**Published:** 2020-03-12

**Authors:** Kelsi A Smith, Sarah Burkill, Ayako Hiyoshi, Tomas Olsson, Shahram Bahmanyar, David Wormser, Yvonne Geissbühler, Alan Moore, Vineetkumar Kharat, Scott Montgomery

**Affiliations:** Clinical Epidemiology Unit, Department of Medicine, Solna, Karolinska Institutet, Stockholm, Sweden/Department of Translational Epidemiology, Institute of Environmental Medicine, Karolinska Institutet, Stockholm, Sweden; Clinical Epidemiology Unit, Department of Medicine, Solna, Karolinska Institutet, Stockholm, Sweden/Centre for Pharmacoepidemiology, Department of Medicine, Solna, Karolinska Institutet, Stockholm, Sweden; Clinical Epidemiology and Biostatistics, School of Medical Sciences, Örebro University, Örebro, Sweden/Department of Public Health Sciences, Stockholm University, Stockholm, Sweden; Department of Clinical Neuroscience, Karolinska Institutet, Stockholm, Sweden/Centre for Molecular Medicine, Karolinska University Hospital, Stockholm, Sweden; Clinical Epidemiology Unit, Department of Medicine, Solna, Karolinska Institutet, Stockholm, Sweden/Centre for Pharmacoepidemiology, Department of Medicine, Solna, Karolinska Institutet, Stockholm, Sweden/Centre for Psychiatry Research, Karolinska Institutet, Stockholm, Sweden; F. Hoffmann-La Roche Ltd., Basel, Switzerland; Novartis Pharma AG, Basel, Switzerland; Novartis Pharma AG, Basel, Switzerland; Novartis Pharmaceuticals Corporation, East Hanover, NJ, USA; Clinical Epidemiology Unit, Department of Medicine, Solna, Karolinska Institutet, Stockholm, Sweden/Clinical Epidemiology and Biostatistics, School of Medical Sciences, Örebro University, Örebro, Sweden/Department of Epidemiology and Public Health, University College London, London, UK

**Keywords:** Multiple sclerosis, comorbidity, prevalence, Sweden, registries, chronic disease, cohort, burden

## Abstract

**Background:**

People with multiple sclerosis (pwMS) have increased comorbid disease (CMD) risk. Most previous studies have not considered overall CMD burden.

**Objective:**

To describe lifetime CMD burden among pwMS.

**Methods:**

PwMS identified using Swedish registers between 1968 and 2012 (*n* = 25,476) were matched by sex, age, and county of residence with general-population comparators (*n* = 251,170). Prevalence, prevalence ratios (PRs), survival functions, and hazard ratios by MS status, age, and time period compared seven CMD: autoimmune, cardiovascular, depression, diabetes, respiratory, renal, and seizures.

**Results:**

The magnitude of the PRs for each CMD and age group decreased across time, with higher PRs in earlier time periods. Before 1990, younger age groups had higher PRs, and after 1990, older age groups had higher PRs. Male pwMS had higher burden compared with females. Overall, renal, respiratory, and seizures had the highest PRs. Before 2001, 50% of pwMS received a first/additional CMD diagnosis 20 years prior to people without MS, which reduced to 4 years after 2001. PwMS had four times higher rates of first/additional diagnoses in earlier time periods, which reduced to less than two times higher in recent time periods compared to people without MS.

**Conclusion:**

Swedish pwMS have increased CMD burden compared with the general population, but this has reduced over time.

## Introduction

People with multiple sclerosis (pwMS) have increased risk of additional diseases including cardiovascular, respiratory, autoimmune, and mood disorders such as depression, among others.^1–5^ These comorbid diseases (CMD), the presence of one or more additional conditions that occur in addition to MS,^6^ are associated with delays in MS diagnosis and initiation of disease-modifying treatments (DMT).^7^ CMD are known to increase both MS relapses and disease severity,^8,9^ providing significant challenges to health-care providers managing patients’ overall clinical profiles^9^ and increases a patient’s burden for their own self-care.^10^ Although pwMS generally have increased contact with health-care services due to their MS, attention may not be focused on diseases other than their MS.^11^ Understanding the CMD burden for pwMS by age, sex, and how it changes over time can identify needs for early detection and screening. Increased surveillance for common CMD may also reduce additional burden faced by health-care systems as multi-morbidity care has substantial medical and financial costs.^6,12^

Despite studies demonstrating increased risk of CMD among pwMS, the prevalence of overall comorbid burden remains largely unknown or is known for specific diseases rather than multiple conditions. To date, no study has provided comprehensive estimates of CMD prevalence by age, sex, and time period of pwMS compared to the general population in Sweden. Therefore, this study aims to describe the lifetime burden of CMD in pwMS compared with a sample of the general population and determine whether pwMS has an overall increased prevalence or risk of CMD diagnoses.

## Methods

### Study population and data sources

This cohort study identified pwMS in Sweden recorded from either the Swedish Multiple Sclerosis Register (MSR) or National Patient Register (NPR) with a primary or secondary diagnosis using International Classification of Diseases (ICD) codes (ICD-10 code G35, ICD-9 or ICD-8 code 340) between 1968 and 2012. The NPR includes inpatient hospital diagnoses since 1964 and outpatient diagnoses since 2001.^13^ Diagnostic accuracy of the NPR for diseases that are not rare such as the disease groups studied here varies between 85% and 95% as described elsewhere.^14^ The MSR introduced in 1996 provides a high degree of diagnostic accuracy and coverage.^15,16^

PwMS (*n* = 29,310) were individually matched by birth year, sex, region, and vital status at earliest date of MS diagnosis (index date) from either the NPR or MSR with up to 10 randomly selected general-population comparators (*n* = 293,094) using the Total Population Register, which includes information on vital status and region of residence of all Swedish residents from 1968 onward. Highest attained education level was obtained from the Longitudinal Integrated Database for Health Insurance and Labour Market Studies (LISA).^17^ Data linkage was achieved using the unique personal-identity number issued to all Swedish residents.

Study entry began from index date (date of first MS diagnosis or the same date for the matched comparators). Individuals were included if alive, had not emigrated from Sweden prior to study entry, and were 6–100 years of age. Matched individuals without MS were excluded if they did not meet the above inclusion criteria. Therefore, 25,476 pwMS and 251,170 matched individuals without MS were included.

### Outcome definitions

Seven chronic CMD categories were considered: autoimmune, cardiovascular, depression, diabetes, respiratory, renal, and seizures. These disease categories were previously identified as being overrepresented in pwMS.^1–3,18,19^ For the general-population cohort, diagnoses may have been a first or a comorbid diagnosis. A CMD diagnosis was defined as a record of any ICD code in the NPR (Supplement 1). Diagnoses prior to and post index date were identified. If individuals had more than one disease diagnosis in the same disease category, they were counted only once using the earliest date of diagnosis and were considered affected for all subsequent years.

### Ethical considerations

Approval was given by the Regional Ethical Review Board at Karolinska Institutet (2013/1156-31/5). Informed consent is required for inclusion in the MSR.

### Statistical analysis

#### Prevalence and prevalence ratios

Prevalence over four time periods, 1968–1980, 1981–1990, 1991–2000, and 2001–2012, was determined in age groups of 6–18, 19–40, 41–60, 61–80, and 81–100 years and stratified by MS status and sex. These time periods were chosen to identify the earliest years of the NPR; the years before (DMT) were available; the years in which the first-line DMT became available; and finally, availability of the second-line DMT in conjunction with the addition of outpatient hospital diagnoses to the NPR. PwMS and the general-population cohort entered the denominator of the age group in each time period provided they had not emigrated or died for all attained age groups within the time period. Individuals could contribute to multiple age groups and time periods. The prevalence numerator was the total number of individuals in the age group and time period with the specific CMD calculated with 95% confidence intervals (CIs). Prevalence ratios (PRs) with 95% CIs comparing pwMS with the general population were calculated.

#### Survival probabilities and hazard ratios

To determine age at diagnosis and rate of a first/additional CMD by MS status, survival probabilities and hazard ratios were calculated stratified by both time period and baseline CMD status. Individuals entered at index date and were at risk until occurrence of their first/additional CMD diagnosis in any of the disease categories: death, emigration, age 100 years, or end of the time period, whichever came first. Provided the individual had valid entry characteristics by the beginning of the next time period, individuals were included again and stratified by their new baseline CMD status in the time period.

Survival probabilities and hazard ratios were predicted using flexible non-parametric survival (FNPS) models with age as the underlying timescale using restricted cubic splines. FNPS allows greater flexibility in the shape of hazard functions compared with other non-parametric models and easily accommodates multiple timescale and time-dependent effects.^20^ Four-knot models placed according to default locations^20^ modeled age as the underlying timescale. Due to violations in the proportional hazards assumption assessed by the Schoenfeld residuals, two internal knots were used to model the (time-varying) effect of MS status by age and baseline CMD status. CMD status was categorized as 0, 1, and 2+ as few individuals had more than two CMD. Visual inspection of hazard functions derived from models and minimization of the Akaike and Bayes Information Criteria determined number of knots.

Standardized survival curves and hazard ratios (HRs) were predicted taking into account differences in characteristics between people with and without MS to ensure comparability of estimates.^21^ Potential confounders included sex, county of residence, educational level (compulsory school education or less, post-compulsory secondary school, post-secondary education, and educational data unavailable), and overall follow-up time split by 2-year intervals.

#### Sensitivity analyses

Two sensitivity analyses were conducted to define possible seizure etiology and type of diabetes. For people classified with seizures in each time period, the proportion of them with one primary diagnosis or two primary diagnoses at two separate occasions were determined as this gives a crude indication of possible chronic seizures. To determine the proportion of people with type 1 and 2 diabetes within each time period, their age at first diabetes diagnosis was used for classification and is further described in Supplement 4. Statistical analysis was performed using SAS software version 9.4^22^ for prevalence estimates and the command stpm2^20^ and stpm2_standsurv were used for the FNPS in Stata 15 software.

## Results

### Baseline characteristics

The proportion of pwMS with incident diagnosis of MS was greater than prevalent diagnosis over several time periods, with exception of 1991–2000 (Table 1). The majority of individuals were females aged 19–40 and 41–60 years. PwMS had more CMD at study entry than the general population in all time periods. Education level was similar among pwMS and the general population cohort.

**Table 1.** Demographic information across time periods by MS status.

	1968–1980		1981–1990		1991–2000		2001–2012	
	MS	Non-MS	MS	Non-MS	MS	Non-MS	MS	Non-MS
Total	5029	50,284	9170	90,099	12,456	121,693	21,897	213,438
Prevalent^a^	39 (0.78)		3988 (43.49)		6814 (54.70)		9836 (44.92)	
Incident^a^	4990 (99.22)		5182 (56.51)		5642 (45.30)		12,061 (55.08)	
Female sex	3054 (60.73)	30,536 (60.73)	5778 (63.01)	56,932 (63.19)	8363 (67.14)	82,236 (67.58)	15,122 (69.06)	147,994 (69.34)
Age at entry (years)								
6–18	67 (1.33)	670 (1.33)	72 (0.79)	720 (0.80)	95 (0.76)	949 (0.78)	341 (1.56)	3385 (1.59)
19–40	1713 (34.06)	17,130 (34.07)	2944 (32.10)	29,207 (32.42)	3581 (28.75)	35,560 (29.22)	9537 (43.55)	93,146 (43.64)
41–60	2193 (43.61)	21,930 (43.61)	3833 (41.80)	37,879 (42.04)	5883 (47.23)	58,063 (47.71)	9432 (43.07)	91,890 (43.05)
61–80	947 (18.83)	9470 (18.83)	2108 (22.99)	20,328 (22.56)	2687 (21.57)	25,376 (20.85)	2413 (11.02)	23,293 (10.91)
81–100	109 (2.17)	1084 (2.16)	213 (2.32)	1965 (2.18)	210 (1.69)	1745 (1.43)	174 (0.79)	1724 (0.81)
Total comorbid diseases at entry								
0	3746 (74.49)	46,769 (93.01)	6446 (70.29)	78,646 (87.29)	8604 (69.08)	100,582 (82.65)	13,952 (63.72)	158,080 (74.06)
1	1007 (20.02)	3013 (5.99)	2053 (22.39)	9480 (10.52)	2874 (23.07)	17,444 (14.33)	5731 (26.17)	43,499 (20.38)
2	231 (4.59)	416 (0.83)	538 (5.87)	1626 (1.80)	779 (6.25)	3026 (2.49)	1655 (7.56)	9300 (4.36)
3+	45 (0.89)	86 (0.17)	133 (1.45)	347 (0.39)	199 (1.60)	641 (0.53)	559 (2.55)	2559 (1.20)
Education								
Compulsory	2278 (45.30)	21,093 (41.95)	4014 (43.77)	36,298 (40.29)	4264 (34.23)	39,368 (32.35)	4889 (22.33)	46,434 (21.76)
Post-compulsory	1219 (24.24)	13,764 (27.37)	2890 (31.52)	29,994 (33.29)	5020 (40.30)	49,011 (40.27)	9737 (44.47)	94,244 (44.16)
Post-secondary	582 (11.57)	6864 (13.65)	1378 (15.03)	16,266 (18.05)	2887 (23.18)	31,114 (25.57)	7102 (32.43)	71,148 (33.33)
Unknown	950 (18.89)	8563 (17.03)	888 (9.68)	7541 (8.37)	285 (2.29)	2200 (1.81)	169 (0.77)	1612 (0.76)

MS: multiple sclerosis; pwMS: people with multiple sclerosis.

a Incident pwMS were ascertained by being a new diagnosis of MS within the time period. Between 1968 and 1980, it is likely that many of these are not incident diagnosis given that the National Patient Register began in 1964 and the start of the study period was 1968. All pwMS in 1968 were prevalent cases; therefore, incident cases between 1968 and 1980 were defined as having an MS diagnosis after 1 January 1969. In other time periods, incident cases had no prior MS diagnosis before the start of the time period. Prevalent cases were defined as having MS at the beginning of the time period.

### Prevalence by time period and sex

**Figure 1.**
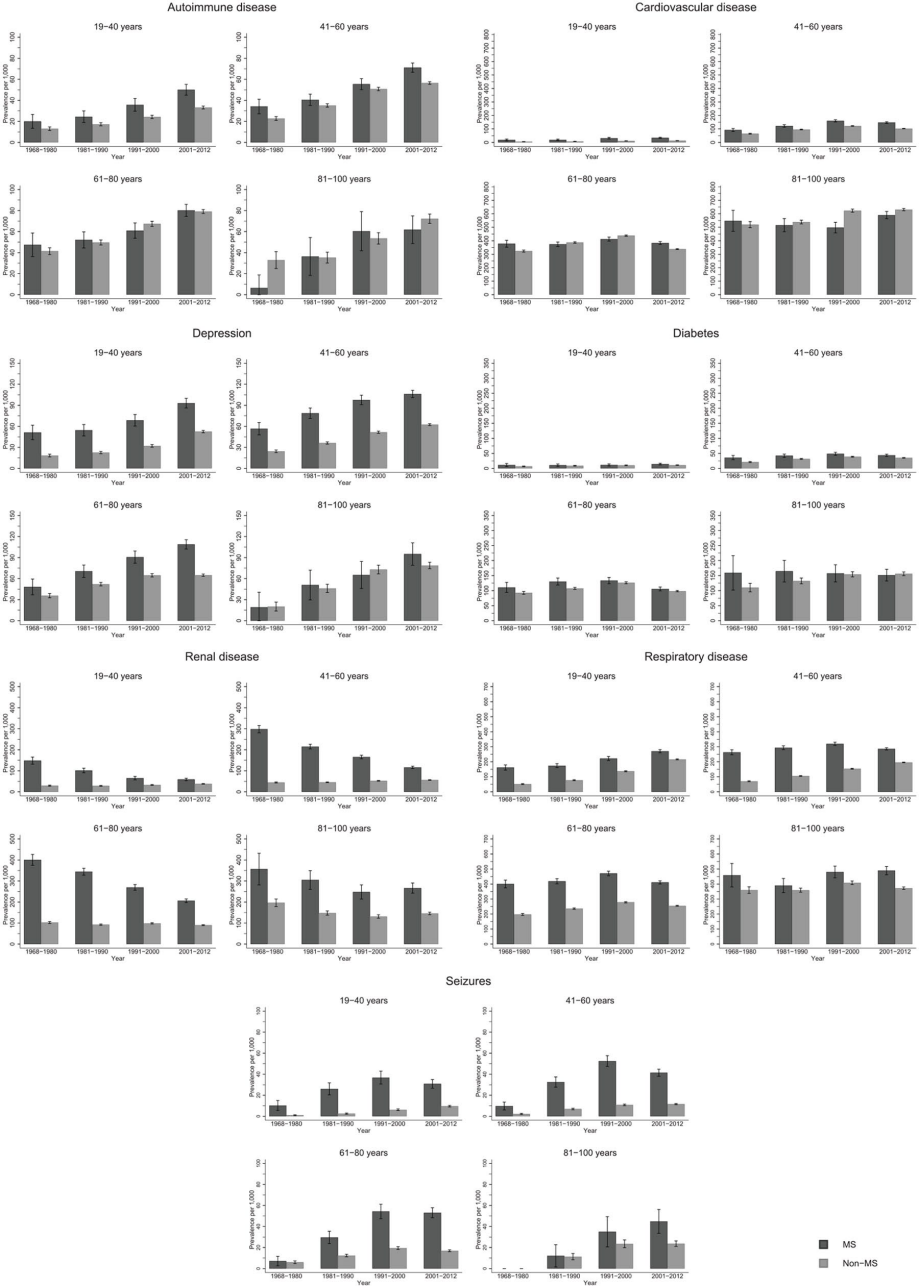
*Overall* prevalence of autoimmune, cardiovascular, depression, diabetes, renal, respiratory and seizure disease among persons with MS and the general population comparators with 95% confidence intervals stratified by time period.

**Figure 2.**
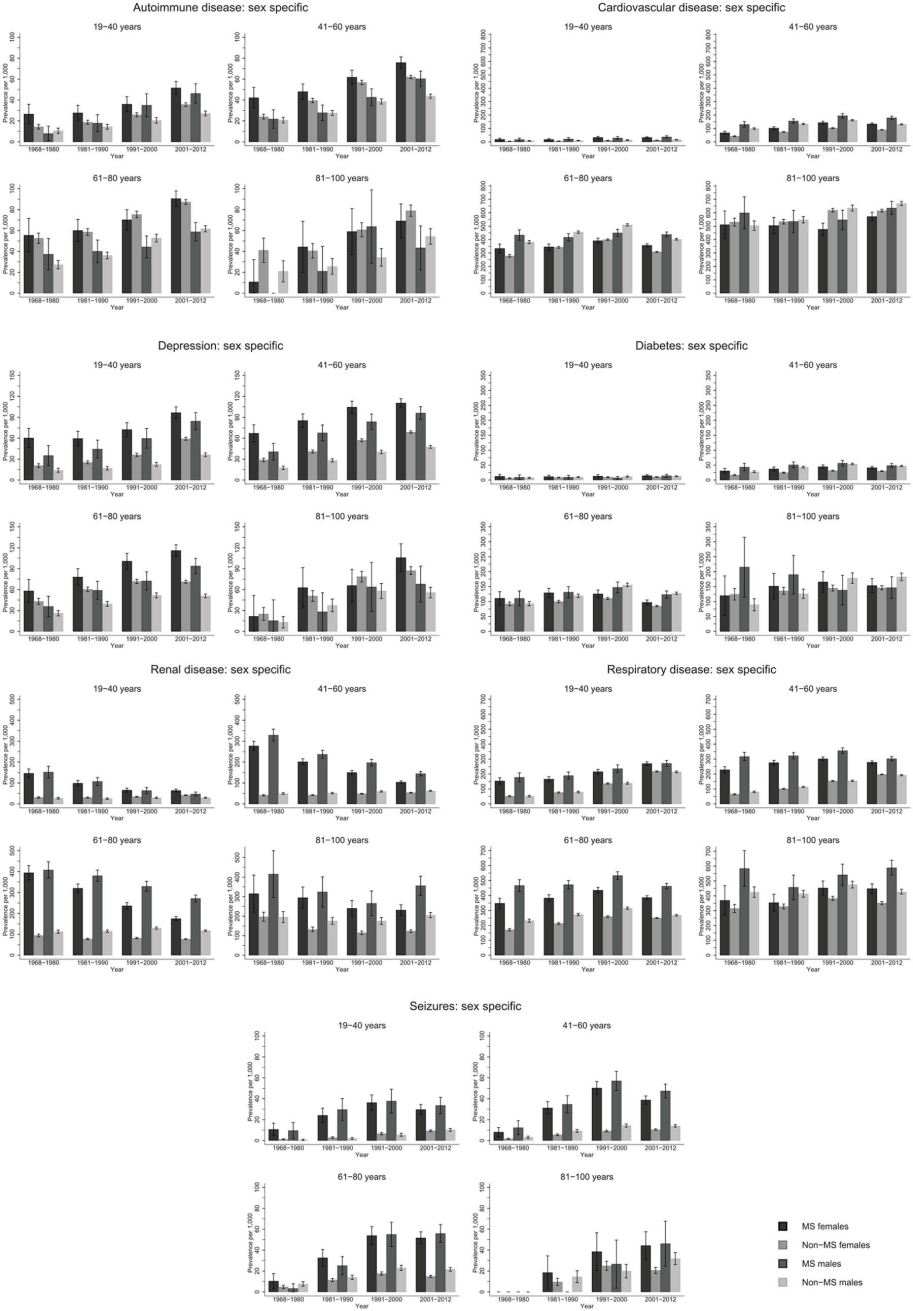
*Sex-specific* prevalence of autoimmune, cardiovascular, depression, diabetes, renal, respiratory and seizure diseases among people with MS and the general population comparators with 95% confidence intervals stratified by time-period.

The prevalence of autoimmune disease and depression increased steeply from 1968–1980 to 2001–2012 in the same age groups (Figure 1; numbers and CIs in Supplement 2). Seizure and respiratory disease prevalence increased moderately over time, while cardiovascular disease (CVD) and diabetes remained stable. Renal disease steeply decreased over time. Similar trends were observed among male and female pwMS (Figure 2). Exceptions to these trends included decrease in diabetes prevalence among male pwMS 81–100 years, increase in respiratory disease prevalence among male and female pwMS 19–40 years, and an increase in prevalence of seizures among both sexes with MS until 2001–2012 where it decreased. The majority of individuals with diabetes had type 2 diabetes (Supplement 4).

### Prevalence by age group and sex

Few pwMS between 6–18 years were diagnosed with CMD: highest prevalence among both sexes was respiratory disease and lowest prevalence was for depression and CVD irrespective of time period and is included in tables in Supplement 2 due to low numbers in this age group. Seizure prevalence was higher for male pwMS (Figure 2), and autoimmune and renal diseases prevalence was higher among female pwMS (Figure 2). Among both sexes 19–40 years with MS, highest prevalence was for respiratory disease and renal disease, although renal disease prevalence decreased over time (Figure 2, Supplement 2). Lowest prevalence was for diabetes and CVD, but autoimmune disease was higher among female pwMS. For both sexes with MS, 41–60 years had highest prevalence for respiratory and renal disease (in earlier time periods) and CVD. Lowest prevalence among female pwMS was seizures and diabetes and autoimmune disease and seizures among male pwMS. Male pwMS had higher prevalence for renal, respiratory, and CVD than females as females had higher prevalence of autoimmune disease and depression. Both sexes aged 61–80 years had highest prevalence of respiratory disease, CVD, and renal disease in earlier time periods, although all were higher among male pwMS. Lowest prevalence for both sexes with MS was autoimmune (lower among females) and seizures. Depression was higher among females. Both sexes with MS 81–100 years had highest prevalence for renal disease (in earlier time periods), CVD, and respiratory disease with men having a much higher prevalence for all of them, including diabetes. Lowest prevalence was autoimmune disease and seizures for both sexes, although women had higher depression prevalence than male pwMS.

### Prevalence ratios (PRs)

**Figure 3.**
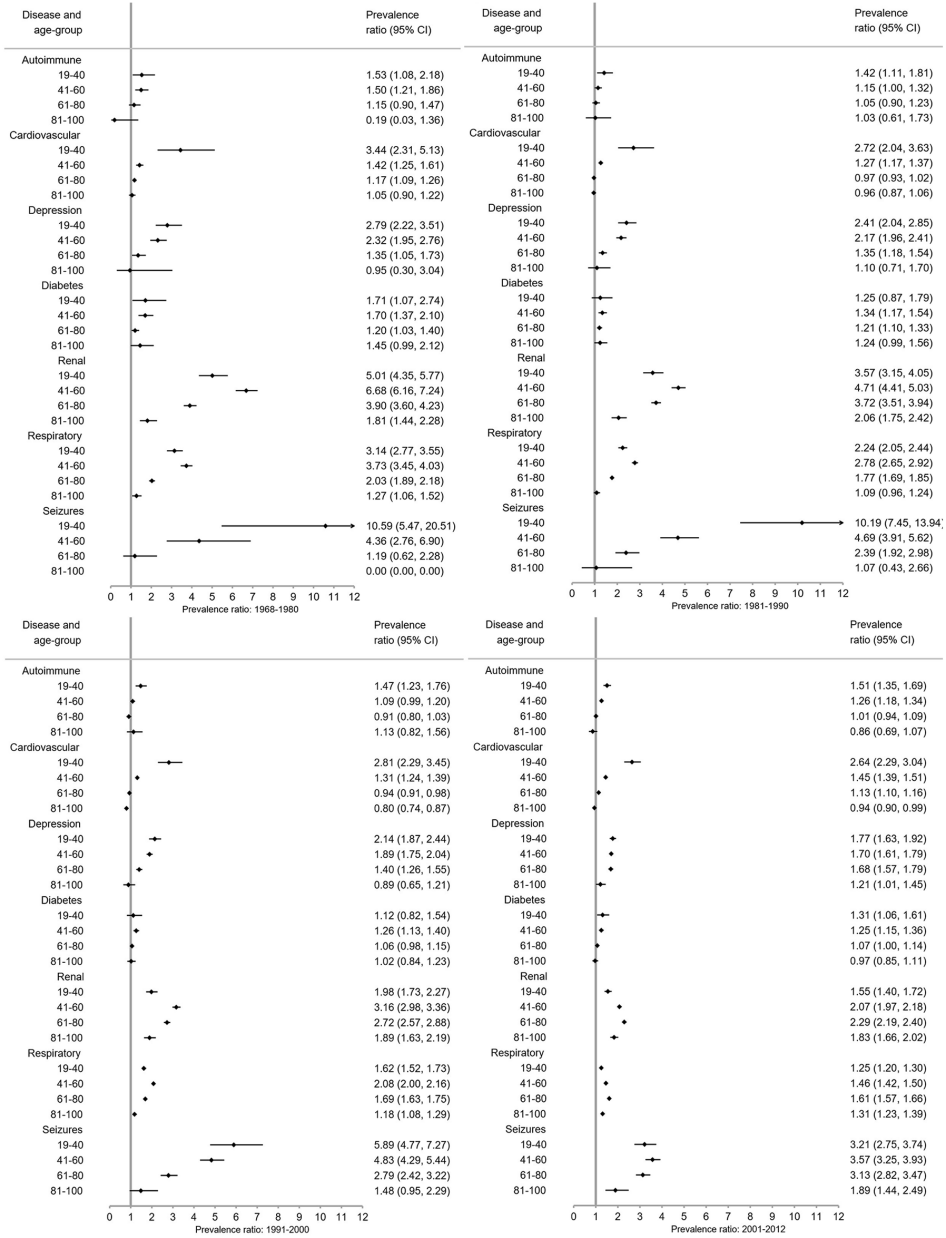
*Overall* prevalence ratios of comorbid diseases among persons with MS compared to the general population with 95% confidence intervals (CI) stratified by time period.

**Figure 4.**
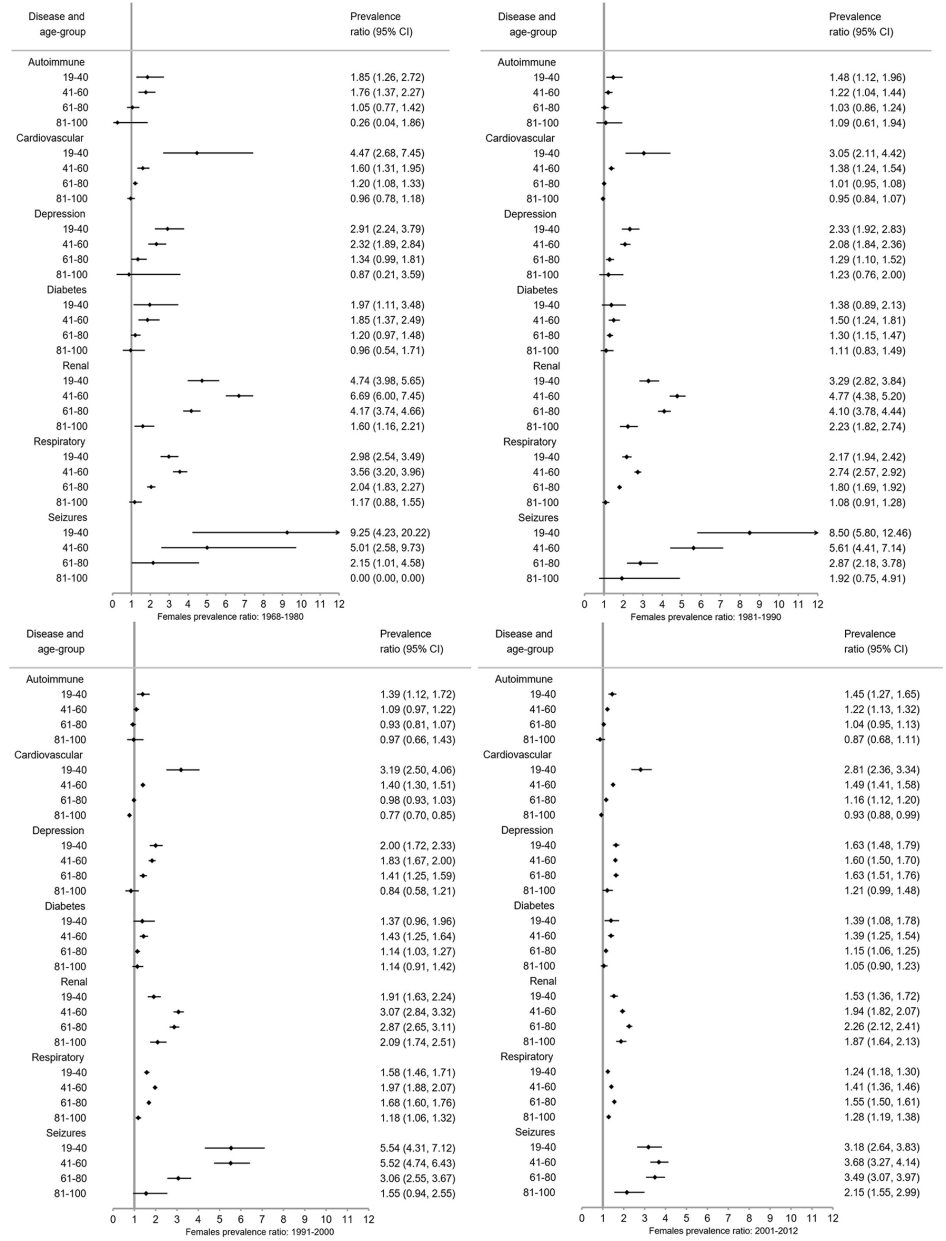
Prevalence ratios of comorbid diseases among *females* with MS compared to the *female* general population with 95% confidence intervals (CI) stratified by time period.

**Figure 5.**
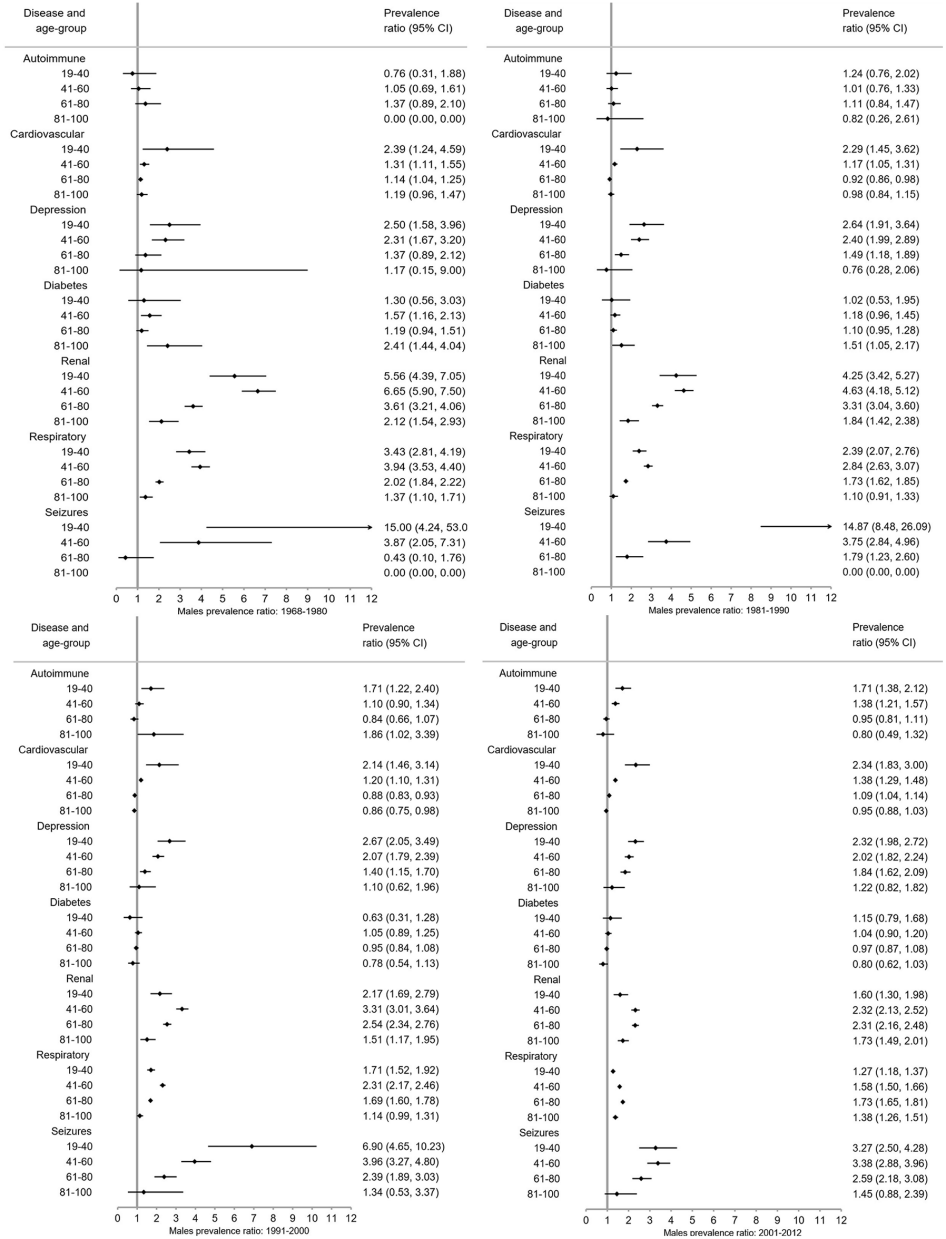
Prevalence ratios of comorbid diseases among *males* with MS compared to the *male* general population with 95% confidence intervals (CI) stratified by time period.

Overall (Figure 3) and for each sex (Figures 4 and 5), pwMS were more burdened with CMD in nearly all age groups (PRs > 1). The magnitude of the PRs for each CMD and age group decreased over time, with higher PRs observed in earlier time periods. PwMS are more similar to the general population especially in 2001–2012 compared to earlier time periods, but PRs remained elevated for seizures and renal disease between 2001–2012. However, renal, respiratory, and seizure diseases were the highest PRs among both male and female pwMS compared to the general population throughout all time periods. Females also had increased PRs for depression.

**Figure 6.**
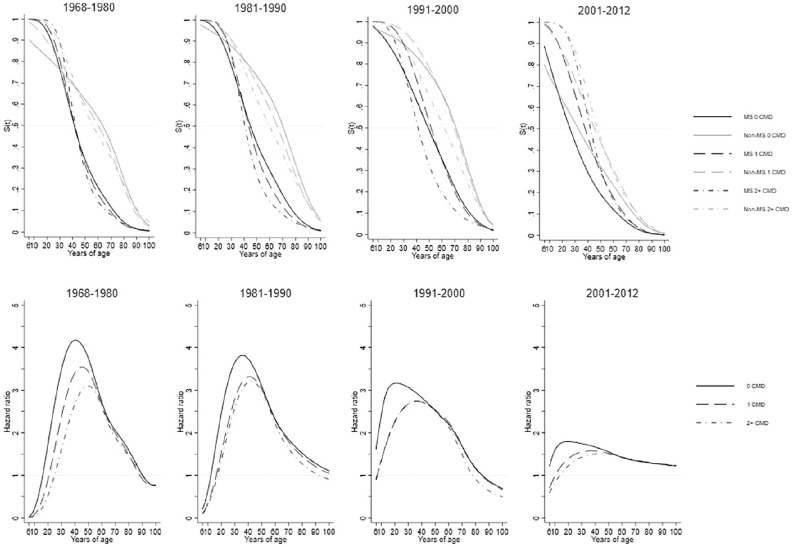
Standardized survival curves (upper panel) and hazard ratios (lower panel) comparing persons with MS to the general population, stratified by baseline number of comorbid diseases at the beginning of each time period. Flexible non-parametric models were used adjusting for sex, county of residence at index date, education, and years since study entry. All seven disease categories were combined and time to the next (first or additional) disease diagnosis from the beginning of the time period was used.

Between 1968–1980 and 1981–1990, pwMS in younger age groups (<41 years) compared to the general population had higher PRs than older age groups with the exception of respiratory and renal diseases (Figures 4–6). Between 1991–2000 and 2001–2012, the CMD burden was lower among younger than older age groups. Few CMD occurred among 6–18-year olds and estimates are absent or CIs wide (Supplement 3). In the 81–100 age group, PRs are smaller with larger CIs than other age groups.

Of the individuals included with seizures, the proportion of them with one or two primary diagnoses of seizures was reduced; however, comparing the PRs of individuals with one or two primary diagnosis remained similar and were similar to the main seizure burden analysis (results not shown). This indicates that the possible seizure etiology is more representative of individuals with repeated seizures (epilepsy) than single episodes.

### Age at diagnosis and hazard ratios

The age at diagnosis for 50% of pwMS for a first/additional CMD diagnosis in all time periods but 2001–2012 was approximately 20 years before the general population irrespective of initial CMD status (Figure 6). In 2001–2012, the difference between pwMS and the general population with 0 baseline CMD diagnoses was approximately 4 years. The proportion of pwMS at younger ages, irrespective of their CMD status in each time period, who developed a first/additional CMD was much greater than the general population who experience their first/additional CMD at older ages. The age range when pwMS had a first/additional diagnosis is between 40 to 52 years, whereas this ranged from 58 to 72 years for the general population cohort. This differed in 2001–2012 ranging for pwMS between 28 to 42 years, and the general-population cohort is between 32 to 50 years. The pwMS and the general population survival curves were most similar when pwMS had 0 baseline CMD and the general population had 2+ baseline CMD, as these were the closest in age when 50% of both groups experienced a first/additional event. That pwMS and the general population became more similar over time was especially reflected in the HRs with higher HRs in earlier time periods. HRs in 1968–1980 exceeded 4.00 at their peak, reducing steadily over time to lower than 2.00 in 2001–2012. However, over the entire life span of pwMS compared to the general population in all time periods (with the exception of <18 and >85 years), pwMS had an increased rate of additional diagnoses, irrespective of their baseline CMD status.

## Discussion

PwMS had higher CMD burden than the general population. This shifted from younger to older age groups over time, but still 50% of pwMS were diagnosed with a first/additional CMD diagnosis 20 years before the general population in all time periods except 2001–2012. Male pwMS had higher burden than females, but for both sexes highest disease burden was for renal, respiratory, and seizure diseases, although there was slight variation by age group. Female pwMS had higher burden of autoimmune disease and depression.

PwMS are known to be at higher risk of CMD. Previously, no studies (we know of) have determined disease burden prevalence or PRs among different age groups, by sex, time period and over a wide variety of disease categories in Sweden using national-level data. Several meta-analyses of CMD in pwMS were recently published indicating a higher proportion of autoimmune diseases,^3^ depression,^2^ various cardiovascular diseases,^1^ diabetes,^1^ epilepsy/seizures,^23^ and lung disease,^4^ consistent with overall trends reported here. However, this study found that PRs of renal disease was higher among pwMS than the general population, despite their prevalence drastically decreasing over time for only pwMS, in contrast to PRs of renal disease reported in other studies.^4^ The most common CMD previously reported among pwMS were depression and chronic lung diseases;^18^ however, this study found that CVD and respiratory and renal diseases were most prevalent, with depression only most common among females.

The magnitude of PRs were lower in 81–100 year olds. This is due to pwMS experiencing shortened life spans, an effect most pronounced in this study’s male MS population. In addition, people with and without MS who live longer may have fewer CMD than people with shorter lives. In addition, CIs were very wide among 6–18 year olds as pediatric MS is uncommon, and they had few CMD. Point estimates among this group may be unreliable and should be interpreted with caution; however; they still provide an indication of disease burden.

Male pwMS had greater disease burden than female, especially for renal, respiratory, and CVD. Females had higher burden of depression and autoimmune disease, although both sexes had a higher burden of CMD than the general population. Seizures had some of the highest PR especially among females with MS, but their prevalence was much lower than other CMD. Females may seek health care at an earlier time possibly reducing the need for hospital care as women have been shown to be older at first hospital admission.^24^ Higher rates in males could be due to the increased severity of their MS requiring hospital care^25^ in particular for respiratory disease as pwMS tend to have more respiratory dysfunction.^26^ MS has a long and insidious latency period, with appearance of symptoms occurring over years. Therefore, pre-clinical symptoms of MS could contribute to development or diagnosis of another chronic disease as pwMS have been shown to have increased health-care utilization 5 years prior to MS onset.^27^ Awareness of CMD is especially important for pwMS, they may interpret symptoms as part of their MS, leading to synergistic negative effects of multiple conditions having an impact on disability,^28^ quality of life,^29^ and risk of hospital admission.^11^

### Implications of CMD and MS

MS treatment typically takes a multi-disciplinary approach to manage the wide breadth of symptoms. A patient’s clinical complexity can be influenced by disease course, also by age, sex and CMD; all of which could influence disease outcomes. This was demonstrated here by the observed sex and age differences for people with and without MS in prevalence and risk of first/additional CMD, although differences over time became less pronounced especially with transition of disease burden from younger to older ages over time. There was also a marked decrease in PRs over time showing a reduced burden among pwMS compared to non-MS. This suggests better management of MS and use of more effective DMT, possibly slowing the progression of MS and accumulation of CMD. Many CMD occurred in pwMS 20 years prior to the general population, but possibly due to effective treatments and more awareness of MS among clinicians and pwMS, the age difference reduced to 4 years in 2001–2012. Although when comparing higher levels of baseline CMD (1,2+) in 2001–2012, the age difference was 8 years, which suggests the possibility of improvement in screening and monitoring for CMD and counseling on modifiable risk factors in pwMS. This may not only reduce the risk of CMD but also have implications for MS and severity due to influence of CMD, particularly among male pwMS.

### Advantages and limitations

Advantages of this study include use of both the MSR and NPR to ensure the widest capture of MS patients, with no loss to follow-up in Sweden between 1968 and 2012. Using both registers allowed use of the earliest possible recorded date of MS diagnosis. Due to the wide-inclusion criteria, the results of this study may be generalizable to other Nordic countries, or other high-income countries with similar standards of hospital-care and patient treatment.

Potential limitations include not being able to control for lifestyle risk factors such as smoking.^30^ Another limitation includes possible misclassification of CMD as the first diagnosis of a CMD was used to identify affected individuals. However, using hospital diagnoses may reduce the number of misclassifications versus primary-care records as hospital diagnoses indicate more severe conditions requiring medical care: this will apply equally to those with and without MS. Although the NPR has good diagnostic accuracy for more common diseases such as CVD and diabetes and some autoimmune diseases like rheumatoid arthritis,^14^ it may be lower for less common diseases or diseases that are less severe not requiring a hospital admission. The specificity of some ICD codes was limited, such as for type 1 and 2 diabetes and chronic or isolated seizures. For diabetes, age at first diagnosis was used to broadly differentiate between the disease types and for seizures we examined single or multiple hospital contacts with seizures as the primary discharge or outpatient diagnosis. While neither of these approaches will be completely accurate, they largely indicate the proportion of disease types and how this differs between people with and without MS. Furthermore, the use of broad disease categories examined the overall disease profile rather than a specific disease, providing an overview of CMD and reducing misclassification. Some CMD may have been underestimated, such as for depression, as inpatient/outpatient hospital diagnoses were used as primary-care records were unavailable. This may mostly affect the non-MS individuals as they may require less hospital care if they do not have any chronic disease. This is relevant to risk of surveillance bias as pwMS tend to encounter medical professionals more frequently than the general population. Stratifying the survival/hazard models by baseline CMD provided control for regular hospital-care contacts, thus reducing the likelihood of differential surveillance bias. Individuals without MS, but with a chronic disease (1 or 2+ CMD), may also require increased contact with health care increasing comparability with pwMS. The estimates are still representative of conditions warranting a hospital diagnosis, demonstrating the greater burden of disease among pwMS. As pwMS generally have increased contact with health-care services, this may provide a unique opportunity for increased screening for common CMD in order to reduce disease burden and further reduce the differences between pwMS and the general population.

In conclusion, this study provides a comprehensive overview of age-specific and sex-specific CMD burden pwMS experience over their entire life course. Clinicians managing pwMS should be aware of this increased disease burden, and efforts for early screening and prevention may be key in reducing CMD later in life.

## Supplemental Material

MSJ910497_supplement_1 – Supplemental material for Comorbid disease burden among MS patients 1968–2012: A Swedish register–based cohort study Click here for additional data file. Supplemental material, MSJ910497_supplement_1 for Comorbid disease burden among MS patients 1968–2012: A Swedish register–based cohort study by Kelsi A Smith, Sarah Burkill, Ayako Hiyoshi, Tomas Olsson, Shahram Bahmanyar, David Wormser, Yvonne Geissbühler, Alan Moore, Vineetkumar Kharat and Scott Montgomery in Multiple Sclerosis Journal

MSJ910497_supplement_2 – Supplemental material for Comorbid disease burden among MS patients 1968–2012: A Swedish register–based cohort study Click here for additional data file. Supplemental material, MSJ910497_supplement_2 for Comorbid disease burden among MS patients 1968–2012: A Swedish register–based cohort study by Kelsi A Smith, Sarah Burkill, Ayako Hiyoshi, Tomas Olsson, Shahram Bahmanyar, David Wormser, Yvonne Geissbühler, Alan Moore, Vineetkumar Kharat and Scott Montgomery in Multiple Sclerosis Journal

MSJ910497_supplement_3 – Supplemental material for Comorbid disease burden among MS patients 1968–2012: A Swedish register–based cohort study Click here for additional data file. Supplemental material, MSJ910497_supplement_3 for Comorbid disease burden among MS patients 1968–2012: A Swedish register–based cohort study by Kelsi A Smith, Sarah Burkill, Ayako Hiyoshi, Tomas Olsson, Shahram Bahmanyar, David Wormser, Yvonne Geissbühler, Alan Moore, Vineetkumar Kharat and Scott Montgomery in Multiple Sclerosis Journal

MSJ910497_supplement_4 – Supplemental material for Comorbid disease burden among MS patients 1968–2012: A Swedish register–based cohort study Click here for additional data file. Supplemental material, MSJ910497_supplement_4 for Comorbid disease burden among MS patients 1968–2012: A Swedish register–based cohort study by Kelsi A Smith, Sarah Burkill, Ayako Hiyoshi, Tomas Olsson, Shahram Bahmanyar, David Wormser, Yvonne Geissbühler, Alan Moore, Vineetkumar Kharat and Scott Montgomery in Multiple Sclerosis Journal
