# Supplementary material for: Comorbid disease burden among MS patients 1968–2012: A Swedish register–based cohort study
Source: Mult Scler. 2020 Mar 12;27(2):268–80. doi: 10.1177/1352458520910497 (PMC7820574; doi:10.1177/1352458520910497)
Supplement: MSJ910497_supplement_1 – Supplemental material for Comorbid disease burden among MS patients 1968–2012: A Swedish register–based cohort study [file MSJ910497_supplement_1.pdf]

Supplement 1 Swedish ICD codes

Table 1: Full list of ICD 8, 9 and 10 codes used for the disease categories

Disease	ICD-8	ICD-9*	ICD-10
**Autoimmune diseases**			
Autoimmune hemolytic anemia	283.90, 283.91	283A	D59.0, D59.1
Autoimmune hepatitis	573.00	571E	K75.4
Basedow-Grave disease	242.00, 242.09	242	E06.5
Crohn's disease and enteritis	563.00	555	K50
Cutaneous lupus erythematosus	695.40	695E	L93
Guillan-Barre syndrome	354.01	357A, 357W	G61.0
Hashimoto's thyroiditis	245.03, 242.20	245C	E06.33
Immune thrombocytopenic purpura	287	287D, 287E, 287F	M31.1
Psoriasis	696.0, 696.1, 696.2	696A, 696B, 696C	L40, L41
Rheumatoid arthritis	712	714	M05, M06
Sarcoidosis	135	135	D86
Scleroderma	701.00, 701.01	701A, 701B, 701C	M34
Sjogren's syndrome	734.90	710C	M35.0
Systemic lupus erythematosus	734.1	710A	M32
Ulcerative colitis	563.10	556	K51
**Cardiovascular diseases**			
Central nervous system vascular diseases	430-438, 347.9	430-438	I60-I69
Vascular hypertensive disorders	400,401,402.99, 403.99, 404.99	401-405	I10-I13, I15
Coronary artery disorders	410-414, 423, 429.99, 458.99	410, 411B, 411C, 411X, 413, 414, 429C, 429F, 429G, 429W	I20-I25
**Depression**	296.0, 296.1, 296.2, 296.3, 298.0, 299.0, 300.4, 307, 790.2	296, 298A, 300E, 308, 311	F31-F34, F38, F39
**Diabetes**	250	250	E10-E14
**Renal disease**	580-599	580-589	N0-N2
**Respiratory disease**	460-519	460-519	J00-J99
**Seizure and epilepsy**	345	345	G40, G41

* Swedish ICD-9 codes use alphanumerical values rather than a decimal point followed by numerical values: A=0; B=1; C=2; D=3; E=4; F=5; G=6; H=7; W=8; X=9.
