# Supplementary material for: Comorbid disease burden among MS patients 1968–2012: A Swedish register–based cohort study
Source: Mult Scler. 2020 Mar 12;27(2):268–80. doi: 10.1177/1352458520910497 (PMC7820574; doi:10.1177/1352458520910497)
Supplement: MSJ910497_supplement_2 – Supplemental material for Comorbid disease burden among MS patients 1968–2012: A Swedish register–based cohort study [file MSJ910497_supplement_2.pdf]

Autoimmune		1968-1980			1981-1990			1991-2000			2001-2012		
**Age group**	**Group**	**n (N)**	**Prev**	**CI**	**n (N)**	**Prev**	**CI**	**n (N)**	**Prev**	**CI**	**n (N)**	**Prev**	**CI**
**6-18**													
**Overall**	**MS**	1 (67)	14.93	0-43.96	2 (72)	27.78	0-65.74	1 (95)	10.53	0-31.05	8 (191)	41.88	13.47-70.30
	**Non-MS**	2 (670)	2.99	0-7.12	3 (720)	4.17	0-8.87	7 (949)	7.38	1.93-12.82	20 (1929)	10.37	5.85-14.89
**Females**	**MS**	1 (48)	20.83	0-61.24	2 (56)	35.71	0-84.32	1 (71)	14.08	0-41.50	5 (136)	36.76	5.14-68.39
	**Non-MS**	2 (480)	4.17	0-9.93	3 (560)	5.36	0-11.40	4 (709)	5.64	0.13-11.16	14 (1369)	10.23	4.90-15.56
**Males**	**MS**	0 (19)	0	0-0	0 (16)	0	0-0	0 (24)	0	0-0	3 (55)	54.55	0-114.56
	**Non-MS**	0 (190)	0	0-0	0 (160)	0	0-0	3 (240)	12.50	0-26.56	6 (560)	10.71	2.19-19.24
**19-40**													
**Overall**	**MS**	35 (1753)	19.97	13.42-26.51	73 (2991)	24.41	18.88-29.94	130 (3642)	35.69	29.67-41.72	344 (6867)	50.09	44.94-55.25
	**Non-MS**	228 (17530)	13.01	11.33-14.68	512 (29677)	17.25	15.77-18.73	876 (36177)	24.21	22.63-25.80	2260 (68146)	33.16	31.82-34.51
**Females**	**MS**	30 (1128)	26.60	17.21-35.99	55 (1982)	27.75	20.52-34.98	92 (2557)	35.98	28.76-43.20	250 (4843)	51.62	45.39-57.85
	**Non-MS**	162 (11280)	14.36	12.17-16.56	368 (19673)	18.71	16.81-20.60	656 (25412)	25.81	23.86-27.76	1713 (48061)	35.64	33.98-37.30
**Males**	**MS**	5 (625)	8.00	1.02-14.98	18 (1009)	17.84	9.67-26.01	38 (1085)	35.02	24.08-45.96	94 (2024)	46.44	37.27-55.61
	**Non-MS**	66 (6250)	10.56	8.03-13.09	144 (10004)	14.39	12.06-16.73	220 (10765)	20.44	17.76-23.11	547 (20085)	27.23	24.98-29.49
**41-60**													
**Overall**	**MS**	91 (2664)	34.16	27.26-41.06	208 (5129)	40.55	35.16-45.95	408 (7352)	55.50	50.26-60.73	957 (13445)	71.18	66.83-75.53
	**Non-MS**	610 (26830)	22.74	20.95-24.52	1796 (51160)	35.11	33.51-36.70	3703 (72849)	50.83	49.24-52.43	7475 (132265)	56.52	55.27-57.76
**Females**	**MS**	68 (1610)	42.24	32.41-52.06	155 (3225)	48.06	40.68-55.44	304 (4915)	61.85	55.12-68.59	709 (9340)	75.91	70.54-81.28
	**Non-MS**	389 (16182)	24.04	21.68-26.40	1274 (32236)	39.52	37.39-41.65	2773 (48775)	56.85	54.80-58.91	5714 (91992)	62.11	60.55-63.67
**Males**	**MS**	23 (1054)	21.82	13.00-30.64	53 (1904)	27.84	20.45-35.23	104 (2437)	42.68	34.65-50.70	248 (4105)	60.41	53.13-67.70
	**Non-MS**	221 (10648)	20.76	18.05-23.46	522 (18924)	27.58	25.25-29.92	930 (24074)	38.63	36.20-41.07	1761 (40273)	43.73	41.73-45.72
**61-80**													
**Overall**	**MS**	66 (1391)	47.45	36.28-58.62	167 (3206)	52.09	44.40-59.78	255 (4183)	60.96	53.71-68.21	690 (8608)	80.16	74.42-85.89
	**Non-MS**	604 (14581)	41.42	38.19-44.66	1614 (32506)	49.65	47.29-52.01	2759 (40963)	67.35	64.93-69.78	6670 (84384)	79.04	77.22-80.86
**Females**	**MS**	43 (776)	55.41	39.32-71.51	116 (1934)	59.98	49.40-70.56	189 (2691)	70.23	60.58-79.89	526 (5817)	90.42	83.05-97.79
	**Non-MS**	428 (8139)	52.59	47.74-57.44	1151 (19684)	58.47	55.20-61.75	2002 (26609)	75.24	72.07-78.41	5015 (57512)	87.20	84.89-89.50
**Males**	**MS**	23 (615)	37.40	22.40-52.39	51 (1272)	40.09	29.31-50.88	66 (1492)	44.24	33.80-54.67	164 (2791)	58.76	50.04-67.49
	**Non-MS**	176 (6442)	27.32	23.34-31.30	463 (12822)	36.11	32.88-39.34	757 (14354)	52.74	49.08-56.39	1655 (26872)	61.59	58.71-64.46
**81-100**													
**Overall**	**MS**	1 (157)	6.37	0-18.81	15 (413)	36.32	18.28-54.36	38 (629)	60.41	41.79-79.03	80 (1294)	61.82	48.70-74.95
	**Non-MS**	62 (1882)	32.94	24.88-41.01	171 (4844)	35.30	30.10-40.50	359 (6704)	53.55	48.16-58.94	956 (13230)	72.26	67.85-76.67
**Females**	**MS**	1 (92)	10.87	0-32.06	12 (271)	44.28	19.79-68.77	26 (441)	58.96	36.97-80.94	64 (926)	69.11	52.78-85.45
	**Non-MS**	46 (1120)	41.07	29.45-52.69	127 (3128)	40.60	33.68-47.52	297 (4893)	60.70	54.01-67.39	760 (9621)	78.99	73.60-84.38
**Males**	**MS**	0 (65)	0	0-0	3 (142)	21.13	0-44.78	12 (188)	63.83	28.89-98.77	16 (368)	43.48	22.64-64.31
	**Non-MS**	16 (762)	21.00	10.82-31.18	44 (1716)	25.64	18.16-33.12	62 (1811)	34.24	25.86-42.61	196 (3609)	54.31	46.91-61.70

Supplement 2 Table 1: Autoimmune disease prevalence and confidence intervals (CI) over time, by sex and age-group. Abbreviations: CI=confidence interval; prev=prevalence; n=total number individuals with disease; N=total number individuals in group.

Cardiovascular		1968-1980			1981-1990			1991-2000			2001-2012		
	Group	n (N)	Prev	CI	n (N)	Prev	CI	n (N)	Prev	CI	n (N)	Prev	CI
**6-18**													
**Overall**	**MS**	0 (67)	0	0-0	2 (72)	27.78	0-65.74	0 (95)	0	0-0	1 (191)	5.24	0-15.47
	**Non-MS**	0 (670)	0	0-0	0 (720)	0	0-0	2 (949)	2.11	0-5.03	1 (1929)	0.52	0-1.53
**Females**	**MS**	0 (48)	0	0-0	0 (56)	0	0-0	0 (71)	0	0-0	1 (136)	7.35	0-21.71
	**Non-MS**	0 (480)	0	0-0	0 (560)	0	0-0	2 (709)	2.82	0-6.72	0 (1369)	0	0-0
**Males**	**MS**	0 (19)	0	0-0	2 (16)	125.00	0-287.05	0 (24)	0	0-0	0 (55)	0	0-0
	**Non-MS**	0 (190)	0	0-0	0 (160)	0	0-0	0 (240)	0	0-0	1 (560)	1.79	0-5.28
**19-40**													
**Overall**	**MS**	32 (1753)	18.25	11.99-24.52	58 (2991)	19.39	14.45-24.33	115 (3642)	31.58	25.90-37.26	234 (6867)	34.08	29.78-38.37
	**Non-MS**	93 (17530)	5.31	4.23-6.38	212 (29677)	7.14	6.19-8.10	406 (36177)	11.22	10.14-12.31	880 (68146)	12.91	12.07-13.76
**Females**	**MS**	21 (1128)	18.62	10.73-26.51	36 (1982)	18.16	12.28-24.04	84 (2557)	32.85	25.94-39.76	159 (4843)	32.83	27.81-37.85
	**Non-MS**	47 (11280)	4.17	2.98-5.36	117 (19673)	5.95	4.87-7.02	262 (25412)	10.31	9.07-11.55	562 (48061)	11.69	10.73-12.65
**Males**	**MS**	11 (625)	17.6	7.29-27.91	22 (1009)	21.80	12.79-30.82	31 (1085)	28.57	18.66-38.48	75 (2024)	37.06	28.83-45.28
	**Non-MS**	46 (6250)	7.36	5.24-9.48	95 (10004)	9.50	7.60-11.40	144 (10765)	13.38	11.21-15.55	318 (20085)	15.83	14.11-17.56
**41-60**													
**Overall**	**MS**	245 (2664)	91.97	0.99-102.94	625 (5129)	121.86	112.90-130.81	1176 (7352)	159.96	151.58-168.34	1984 (13445)	147.56	141.57-153.56
	**Non-MS**	1733 (26830)	64.59	61.65-67.53	4898 (51160)	95.74	93.19-98.29	8878 (72849)	121.87	119.49-124.24	13499 (132265)	102.06	100.43-103.69
**Females**	**MS**	108 (1610)	67.08	54.86-79.30	329 (3225)	102.02	91.57-112.46	703 (4915)	143.03	133.24-152.82	1246 (9340)	133.40	126.51-140.30
	**Non-MS**	679 (16182)	41.96	38.87-45.05	2379 (32236)	73.80	70.95-76.65	4998 (48775)	102.47	99.78-105.16	8261 (91992)	89.80	87.95-91.65
**Males**	**MS**	137 (1054)	129.98	109.68-150.28	296 (1904)	155.46	139.19-171.74	473 (2437)	194.09	178.39-209.79	738 (4105)	179.78	168.03-191.53
	**Non-MS**	1054 (10648)	98.99	93.31-104.66	2519 (18924)	133.11	128.27-137.95	3880 (24074)	161.17	156.52-165.81	5238 (40273)	130.06	126.78-133.35
**61-80**													
**Overall**	**MS**	526 (1391)	378.15	352.66-403.63	1200 (3206)	374.30	357.55-391.05	1726 (4183)	412.62	397.70-427.54	3300 (8608)	383.36	373.09-393.64
	**Non-MS**	4720 (14581)	323.71	316.11-331.30	12590 (32506)	387.31	382.02-392.61	17953 (40963)	438.27	433.47-443.08	28538 (84384)	338.19	335.00-341.38
**Females**	**MS**	259 (776)	333.76	300.58-366.94	669 (1934)	345.92	324.72-367.11	1054 (2691)	391.68	373.23-410.12	2076 (5817)	356.88	344.57-369.20
	**Non-MS**	2266 (8139)	278.41	268.67-288.15	6750 (19684)	342.92	336.29-349.55	10636 (26609)	399.71	393.83-405.60	17732 (57512)	308.32	304.54-312.09
**Males**	**MS**	267 (615)	434.15	394.97-473.32	531 (1272)	417.45	390.35-444.55	672 (1492)	450.40	425.16-475.65	1224 (2791)	438.55	420.14-456.96
	**Non-MS**	526 (1391)	378.15	352.66-403.63	1200 (3206)	374.30	357.55-391.05	1726 (4183)	412.62	397.70-427.54	3300 (8608)	383.36	373.09-393.64
**81-100**													
**Overall**	**MS**	2454 (6442)	380.94	369.08-392.80	5840 (12822)	455.47	446.85-464.09	7317 (14354)	509.75	501.58-517.93	10806 (26872)	402.13	396.27-407.99
	**Non-MS**	86 (157)	547.77	469.92-625.63	213 (413)	515.74	467.54-563.94	313 (629)	497.62	458.54-536.69	764 (1294)	590.42	563.62-617.21
**Females**	**MS**	978 (1882)	519.66	497.09-542.23	2610 (4844)	538.81	524.77-552.85	4178 (6704)	623.21	611.61-634.81	8345 (13230)	630.76	622.54-638.99
	**Non-MS**	47 (92)	510.87	408.72-613.02	137 (271)	505.54	446.01-565.06	210 (441)	476.19	429.58-522.80	530 (926)	572.35	540.49-604.22
**Males**	**MS**	594 (1120)	530.36	501.13-559.59	1670 (3128)	533.89	516.41-551.37	3027 (4893)	618.64	605.03-632.25	5929 (9621)	616.26	606.54-625.97
	**Non-MS**	39 (65)	600.00	480.90-719.10	76 (142)	535.21	453.18-617.25	103 (188)	547.87	476.73-619.02	234 (368)	635.87	586.71-685.03

Supplement 2 Table 2: Cardiovascular disease (CVD) prevalence and confidence intervals over time, by sex and age-group. Abbreviations: CI=confidence interval; prev=prevalence; n=total number individuals with disease; N=total number individuals in group.

Depression		1968-1980			1981-1990			1991-2000			2001-2012		
	Group	n (N)	Prev	CI	n (N)	Prev	CI	n (N)	Prev	CI	n (N)	Prev	CI
**6-18**													
**Overall**	MS	0 (67)	0	0-0	1 (72)	13.89	0-40.92	0 (95)	0	0-0	6 (191)	31.41	6.68-56.15
	Non-MS	3 (670)	4.48	0-9.53	1 (720)	1.39	0-4.11	3 (949)	3.16	0-6.73	42 (1929)	21.77	15.26-28.29
**Females**	MS	0 (48)	0	0-0	1 (56)	17.86	0-52.54	0 (71)	0	0-0	4 (136)	29.41	1.02-57.81
	Non-MS	1 (480)	2.08	0-6.16	0 (560)	0	0-0	2 (709)	2.82	0-6.72	33 (1369)	24.11	15.98-32.23
**Males**	MS	0 (19)	0	0-0	0 (16)	0	0-0	0 (24)	0	0-0	2 (55)	36.36	0-85.84
	Non-MS	2 (190)	10.53	0-25.04	1 (160)	6.25	0-18.46	1 (240)	4.17	0-12.32	9 (560)	16.07	5.66-26.49
**19-40**													
**Overall**	MS	90 (1753)	51.34	41.01-61.67	163 (2991)	54.50	46.36-62.63	250 (3642)	68.64	60.43-76.86	639 (6867)	93.05	86.18-99.92
	Non-MS	322 (17530)	18.37	16.38-20.36	671 (29677)	22.61	20.92-24.30	1161 (36177)	32.09	30.28-33.91	3578 (68146)	52.50	50.83-54.18
**Females**	MS	68 (1128)	60.28	46.39-74.17	118 (1982)	59.54	49.12-69.95	185 (2557)	72.35	62.31-82.39	468 (4843)	96.63	88.31-104.96
	Non-MS	234 (11280)	20.74	18.11-23.37	502 (19673)	25.52	23.31-27.72	919 (25412)	36.16	33.87-38.46	2848 (48061)	59.26	57.15-61.37
**Males**	MS	22 (625)	35.20	20.75-49.65	45 (1009)	44.60	31.86-57.34	65 (1085)	59.91	45.79-74.03	171 (2024)	84.49	72.37-96.60
	Non-MS	88 (6250)	14.08	11.16-17.00	169 (10004)	16.89	14.37-19.42	242 (10765)	22.48	19.68-25.28	730 (20085)	36.35	33.76-38.93
**41-60**													
**Overall**	MS	151 (2664)	56.68	47.90-65.46	404 (5129)	78.77	71.40-86.14	718 (7352)	97.66	90.87-104.45	1427 (13445)	106.14	100.93-111.34
	Non-MS	655 (26830)	24.41	22.57-26.26	1854 (51160)	36.24	34.62-37.86	3757 (72849)	51.57	49.97-53.18	8255 (132265)	62.41	61.11-63.72
**Females**	MS	108 (1610)	67.08	54.86-79.30	275 (3225)	85.27	75.63-94.91	514 (4915)	104.58	96.02-113.13	1032 (9340)	110.49	104.13-116.85
	Non-MS	467 (16182)	28.86	26.28-31.44	1320 (32236)	40.95	38.78-43.11	2784 (48775)	57.08	55.02-59.14	6338 (91992)	68.90	67.26-70.53
**Males**	MS	43 (1054)	40.80	28.85-52.74	129 (1904)	67.75	56.46-79.04	204 (2437)	83.71	72.71-94.71	395 (4105)	96.22	87.20-105.25
	Non-MS	188 (10648)	17.66	15.15-20.16	534 (18924)	28.22	25.86-30.58	973 (24074)	40.42	37.93-42.9	1917 (40273)	47.60	45.52-49.68
**61-80**													
**Overall**	MS	67 (1391)	48.17	36.91-59.42	226 (3206)	70.49	61.63-79.35	379 (4183)	90.60	81.91-99.30	937 (8608)	108.85	102.27-115.43
	Non-MS	519 (14581)	35.59	32.59-38.60	1693 (32506)	52.08	49.67-54.50	2648 (40963)	64.64	62.26-67.02	5471 (84384)	64.83	63.17-66.50
**Females**	MS	45 (776)	57.99	41.54-74.43	151 (1934)	78.08	66.12-90.03	271 (2691)	100.71	89.34-112.08	675 (5817)	116.04	107.81-124.27
	Non-MS	351 (8139)	43.13	38.71-47.54	1187 (19684)	60.30	56.98-63.63	1906 (26609)	71.63	68.53-74.73	4101 (57512)	71.31	69.20-73.41
**Males**	MS	22 (615)	35.77	21.09-50.45	75 (1272)	58.96	46.02-71.91	108 (1492)	72.39	59.24-85.53	262 (2791)	93.87	83.05-104.69
	Non-MS	168 (6442)	26.08	22.19-29.97	506 (12822)	39.46	36.09-42.83	742 (14354)	51.69	48.07-55.32	1370 (26872)	50.98	48.35-53.61
**81-100**													
**Overall**	MS	3 (157)	19.11	0-40.52	21 (413)	50.85	29.66-72.04	41 (629)	65.18	45.89-84.47	123 (1294)	95.05	79.07-111.03
	Non-MS	38 (1882)	20.19	13.84-26.55	223 (4844)	46.04	40.13-51.94	489 (6704)	72.94	66.72-79.17	1041 (13230)	78.68	74.10-83.27
**Females**	MS	2 (92)	21.74	0-51.54	17 (271)	62.73	33.86-91.60	29 (441)	65.76	42.63-88.89	98 (926)	105.83	86.02-125.65
	Non-MS	28 (1120)	25.00	15.86-34.14	159 (3128)	50.83	43.13-58.53	384 (4893)	78.48	70.94-86.01	840 (9621)	87.31	81.67-92.95
**Males**	MS	1 (65)	15.38	0-45.31	4 (142)	28.17	0.95-55.38	12 (188)	63.83	28.89-98.77	25 (368)	67.93	42.22-93.64
	Non-MS	10 (762)	13.12	5.04-21.20	64 (1716)	37.30	28.33-46.26	105 (1811)	57.98	47.22-68.74	201 (3609)	55.69	48.21-63.18

Supplement 2 Table 3: Depression prevalence and confidence intervals over time, by sex and age-group. Abbreviations: CI=confidence interval; prev=prevalence; n=total number individuals with disease; N=total number individuals in group.

Diabetes		1968-1980			1981-1990			1991-2000			2001-2012		
	Group	n (N)	Prev	CI	n (N)	Prev	CI	n (N)	Prev	CI	n (N)	Prev	CI
**6-18**													
**Overall**	MS	1 (67)	14.93	0-43.96	0 (72)	0	0-0	1 (95)	10.53	0-31.05	3 (191)	15.71	0-33.34
	Non-MS	0 (670)	0	0-0	3 (720)	4.17	0-8.87	2 (949)	2.11	0-5.03	8 (1929)	4.15	1.28-7.02
**Females**	MS	1 (48)	20.83	0-61.24	0 (56)	0	0-0	1 (71)	14.08	0-41.50	2 (136)	14.71	0-34.94
	Non-MS	0 (480)	0	0-0	2 (560)	3.57	0-8.51	1 (709)	1.41	0-4.17	6 (1369)	4.38	0.88-7.88
**Males**	MS	0 (19)	0	0-0	0 (16)	0	0-0	0 (24)	0	0-0	1 (55)	18.18	0-53.49
	Non-MS	0 (190)	0	0-0	1 (160)	6.25	0-18.46	1 (240)	4.17	0-12.32	2 (560)	3.57	0-8.51
**19-40**													
**Overall**	MS	20 (1753)	11.41	6.44-16.38	33 (2991)	11.03	7.29-14.78	42 (3642)	11.53	8.06-15.00	99 (6867)	14.42	11.60-17.24
	Non-MS	117 (17530)	6.67	5.47-7.88	262 (29677)	8.83	7.76-9.89	372 (36177)	10.28	9.24-11.32	750 (68146)	11.01	10.22-11.79
**Females**	MS	14 (1128)	12.41	5.95-18.87	23 (1982)	11.60	6.89-16.32	34 (2557)	13.30	8.86-17.74	70 (4843)	14.45	11.09-17.82
	Non-MS	71 (11280)	6.29	4.83-7.75	165 (19673)	8.39	7.11-9.66	246 (25412)	9.68	8.48-10.88	500 (48061)	10.40	9.50-11.31
**Males**	MS	6 (625)	9.60	1.96-17.24	10 (1009)	9.91	3.80-16.02	8 (1085)	7.37	2.28-12.46	29 (2024)	14.33	9.15-19.51
	Non-MS	46 (6250)	7.36	5.24-9.48	97 (10004)	9.70	7.78-11.62	126 (10765)	11.70	9.67-13.74	250 (20085)	12.45	10.91-13.98
**41-60**													
**Overall**	MS	96 (2664)	36.04	28.96-43.11	217 (5129)	42.31	36.80-47.82	359 (7352)	48.83	43.90-53.76	589 (13445)	43.81	40.35-47.27
	Non-MS	568 (26830)	21.17	19.45-22.89	1621 (51160)	31.68	30.17-33.20	2832 (72849)	38.87	37.47-40.28	4651 (132265)	35.16	34.17-36.16
**Females**	MS	50 (1610)	31.06	22.58-39.53	120 (3225)	37.21	30.68-43.74	221 (4915)	44.96	39.17-50.76	388 (9340)	41.54	37.49-45.59
	Non-MS	272 (16182)	16.81	14.83-18.79	801 (32236)	24.85	23.15-26.55	1533 (48775)	31.43	29.88-32.98	2756 (91992)	29.96	28.86-31.06
**Males**	MS	46 (1054)	43.64	31.31-55.98	97 (1904)	50.95	41.07-60.82	138 (2437)	56.63	47.45-65.80	201 (4105)	48.96	42.36-55.57
	Non-MS	296 (10648)	27.80	24.68-30.92	820 (18924)	43.33	40.43-46.23	1299 (24074)	53.96	51.10-56.81	1895 (40273)	47.05	44.99-49.12
**61-80**													
**Overall**	MS	154 (1391)	110.71	94.22-127.20	417 (3206)	130.07	118.42-141.71	558 (4183)	133.40	123.09-143.70	910 (8608)	105.72	99.22-112.21
	Non-MS	1349 (14581)	92.52	87.81-97.22	3491 (32506)	107.40	104.03-110.76	5172 (40963)	126.26	123.04-129.48	8302 (84384)	98.38	96.37-100.39
**Females**	MS	86 (776)	110.82	88.74-132.91	250 (1934)	129.27	114.31-144.22	338 (2691)	125.60	113.08-138.13	566 (5817)	97.30	89.68-104.92
	Non-MS	750 (8139)	92.15	85.87-98.43	1962 (19684)	99.67	95.49-103.86	2937 (26609)	110.38	106.61-114.14	4880 (57512)	84.85	82.57-87.13
**Males**	MS	68 (615)	110.57	85.78-135.35	167 (1272)	131.29	112.73-149.85	220 (1492)	147.45	129.46-165.44	344 (2791)	123.25	111.06-135.45
	Non-MS	599 (6442)	92.98	85.89-100.08	1529 (12822)	119.25	113.64-124.86	2235 (14354)	155.71	149.77-161.64	3422 (26872)	127.34	123.36-131.33
**81-100**													
**Overall**	MS	25 (157)	159.24	102.00-216.47	68 (413)	164.65	128.88-200.42	99 (629)	157.39	128.93-185.85	196 (1294)	151.47	131.93-171.00
	Non-MS	207 (1882)	109.99	95.85-124.13	641 (4844)	132.33	122.79-141.87	1033 (6704)	154.09	145.44-162.73	2064 (13230)	156.01	149.83-162.19
**Females**	MS	11 (92)	119.57	53.27-185.87	41 (271)	151.29	108.63-193.96	73 (441)	165.53	130.84-200.22	142 (926)	153.35	130.14-176.56
	Non-MS	139 (1120)	124.11	104.80-143.42	425 (3128)	135.87	123.86-147.88	710 (4893)	145.11	135.24-154.97	1405 (9621)	146.03	138.98-153.09
**Males**	MS	14 (65)	215.38	115.45-315.32	27 (142)	190.14	125.60-254.68	26 (188)	138.30	88.95-187.65	54 (368)	146.74	110.59-182.89
	Non-MS	68 (762)	89.24	69.00-109.48	216 (1716)	125.87	110.18-141.57	323 (1811)	178.35	160.72-195.99	659 (3609)	182.60	169.99-195.20

Supplement 2 Table 4: Diabetes prevalence and confidence intervals over time, by sex and age-group. Abbreviations: CI=confidence interval; prev=prevalence; n=total number individuals with disease; N=total number individuals in group.

Renal		1968-1980			1981-1990			1991-2000			2001-2012		
	Group	n (N)	Prev	CI	n (N)	Prev	CI	n (N)	Prev	CI	n (N)	Prev	CI
**6-18**													
**Overall**	**MS**	5 (67)	74.63	11.70-137.55	4 (72)	55.56	2.65-108.47	3 (95)	31.58	0-66.75	2 (191)	10.47	0-24.91
	**Non-MS**	10 (670)	14.93	5.74-24.11	18 (720)	25.00	13.60-36.40	15 (949)	15.81	7.87-23.74	25 (1929)	12.96	7.91-18.01
**Females**	**MS**	4 (48)	83.33	5.14-161.52	4 (56)	71.43	3.97-138.88	3 (71)	42.25	0-89.05	2 (136)	14.71	0-34.94
	**Non-MS**	8 (480)	16.67	5.21-28.12	15 (560)	26.79	13.41-40.16	13 (709)	18.34	8.46-28.21	21 (1369)	15.34	8.83-21.85
**Males**	**MS**	1 (19)	52.63	0-153.04	0 (16)	0	0-0	0 (24)	0	0-0	0 (55)	0	0-0
	**Non-MS**	2 (190)	10.53	0-25.04	3 (160)	18.75	0-39.77	2 (240)	8.33	0-19.83	4 (560)	7.14	0.17-14.12
**19-40**													
**Overall**	**MS**	260 (1753)	148.32	131.68-164.96	304 (2991)	101.64	90.81-112.47	237 (3642)	65.07	57.06-73.08	405 (6867)	58.98	53.41-64.55
	**Non-MS**	519 (17530)	29.61	27.10-32.12	844 (29677)	28.44	26.55-30.33	1189 (36177)	32.87	31.03-34.70	2595 (68146)	38.08	36.64-39.52
**Females**	**MS**	165 (1128)	146.28	125.65-166.90	196 (1982)	98.89	85.75-112.03	168 (2557)	65.70	56.10-75.31	309 (4843)	63.80	56.92-70.69
	**Non-MS**	348 (11280)	30.85	27.66-34.04	592 (19673)	30.09	27.70-32.48	874 (25412)	34.39	32.15-36.63	2001 (48061)	41.63	39.85-43.42
**Males**	**MS**	95 (625)	152.00	123.85-180.15	108 (1009)	107.04	87.96-126.11	69 (1085)	63.59	49.07-78.12	96 (2024)	47.43	38.17-56.69
	**Non-MS**	171 (6250)	27.36	23.32-31.40	252 (10004)	25.19	22.12-28.26	315 (10765)	29.26	26.08-32.45	594 (20085)	29.57	27.23-31.92
**41-60**													
**Overall**	**MS**	794 (2664)	298.05	280.68-315.42	1103 (5129)	215.05	203.81-226.3	1217 (7352)	165.53	157.04-174.03	1563 (13445)	116.25	110.83-121.67
	**Non-MS**	1198 (26830)	44.65	42.18-47.12	2334 (51160)	45.62	43.81-47.43	3813 (72849)	52.34	50.72-53.96	7441 (132265)	56.26	55.02-57.50
**Females**	**MS**	447 (1610)	277.64	255.76-299.52	651 (3225)	201.86	188.01-215.71	736 (4915)	149.75	139.77-159.72	970 (9340)	103.85	97.67-110.04
	**Non-MS**	671 (16182)	41.47	38.39-44.54	1363 (32236)	42.28	40.09-44.48	2378 (48775)	48.75	46.84-50.67	4929 (91992)	53.58	52.13-55.04
**Males**	**MS**	347 (1054)	329.22	300.85-357.59	452 (1904)	237.39	218.28-256.51	481 (2437)	197.37	181.57-213.18	593 (4105)	144.46	133.7-155.21
	**Non-MS**	527 (10648)	49.49	45.37-53.61	971 (18924)	51.31	48.17-54.45	1435 (24074)	59.61	56.62-62.60	2512 (40273)	62.37	60.01-64.74
**61-80**													
**Overall**	**MS**	557 (1391)	400.43	374.68-426.18	1103 (3206)	344.04	327.60-360.49	1128 (4183)	269.66	256.21-283.11	1777 (8608)	206.44	197.89-214.99
	**Non-MS**	1498 (14581)	102.74	97.81-107.66	3008 (32506)	92.54	89.39-95.69	4054 (40963)	98.97	96.08-101.86	7611 (84384)	90.19	88.26-92.13
**Females**	**MS**	306 (776)	394.33	359.94-428.72	619 (1934)	320.06	299.27-340.85	636 (2691)	236.34	220.29-252.40	1019 (5817)	175.18	165.41-184.94
	**Non-MS**	770 (8139)	94.61	88.25-100.96	1535 (19684)	77.98	74.24-81.73	2188 (26609)	82.23	78.93-85.53	4455 (57512)	77.46	75.28-79.65
**Males**	**MS**	251 (615)	408.13	369.29-446.97	484 (1272)	380.50	353.82-407.18	492 (1492)	329.76	305.90-353.61	758 (2791)	271.59	255.09-288.09
	**Non-MS**	728 (6442)	113.01	105.28-120.74	1473 (12822)	114.88	109.36-120.40	1866 (14354)	130.00	124.50-135.50	3156 (26872)	117.45	113.60-121.30
**81-100**													
**Overall**	**MS**	56 (157)	356.69	281.76-431.62	126 (413)	305.08	260.68-349.49	156 (629)	248.01	214.26-281.76	345 (1294)	266.62	242.52-290.71
	**Non-MS**	370 (1882)	196.60	178.64-214.56	716 (4844)	147.81	137.82-157.81	881 (6704)	131.41	123.33-139.50	1929 (13230)	145.80	139.79-151.82
**Females**	**MS**	29 (92)	315.22	220.28-410.16	80 (271)	295.20	240.89-349.51	106 (441)	240.36	200.48-280.24	214 (926)	231.10	203.95-258.25
	**Non-MS**	221 (1120)	197.32	174.01-220.63	414 (3128)	132.35	120.48-144.23	563 (4893)	115.06	106.12-124.00	1187 (9621)	123.38	116.80-129.95
**Males**	**MS**	27 (65)	415.38	295.58-535.19	46 (142)	323.94	246.97-400.92	50 (188)	265.96	202.80-329.12	131 (368)	355.98	307.06-404.90
	**Non-MS**	149 (762)	195.54	167.38-223.70	302 (1716)	175.99	157.97-194.01	318 (1811)	175.59	158.07-193.12	742 (3609)	205.60	192.41-218.78

Supplement 2 Table 5: Renal disease prevalence and confidence intervals over time, by sex and age-group. Abbreviations: CI=confidence interval; prev=prevalence; n=total number individuals with disease; N=total number individuals in group.

Respiratory		1968-1980			1981-1990			1991-2000			2001-2012		
	Group	n (N)	Prev	CI	n (N)	Prev	CI	n (N)	Prev	CI	n (N)	Prev	CI
**6-18**													
**Overall**	**MS**	3 (67)	44.78	0-94.30	18 (72)	250.00	149.98-350.02	21 (95)	221.05	137.61-304.50	58 (191)	303.66	238.45-368.88
	**Non-MS**	35 (670)	52.24	35.39-69.09	53 (720)	73.61	54.54-92.69	151 (949)	159.11	135.84-182.39	496 (1929)	257.13	237.62-276.63
**Females**	**MS**	3 (48)	62.50	0-130.98	15 (56)	267.86	151.87-383.84	18 (71)	253.52	152.33-354.71	44 (136)	323.53	244.90-402.16
	**Non-MS**	27 (480)	56.25	35.64-76.86	42 (560)	75.00	53.18-96.82	103 (709)	145.28	119.34-171.21	338 (1369)	246.90	224.05-269.74
**Males**	**MS**	0 (19)	0	0-0	3 (16)	187.50	0-378.75	3 (24)	125.00	0-257.32	14 (55)	254.55	139.42-369.67
	**Non-MS**	8 (190)	42.11	13.55-70.66	11 (160)	68.75	29.54-107.96	48 (240)	200.00	149.39-250.61	158 (560)	282.14	244.87-319.42
**19-40**													
**Overall**	**MS**	284 (1753)	162.01	144.76-179.26	520 (2991)	173.85	160.27-187.44	807 (3642)	221.58	208.09-235.07	1856 (6867)	270.28	259.77-280.78
	**Non-MS**	905 (17530)	51.63	48.35-54.90	2301 (29677)	77.53	74.49-80.58	4955 (36177)	136.97	133.42-140.51	14725 (68146)	216.08	212.99-219.17
**Females**	**MS**	173 (1128)	153.37	132.34-174.40	329 (1982)	165.99	149.61-182.37	551 (2557)	215.49	199.55-231.42	1307 (4843)	269.87	257.37-282.38
	**Non-MS**	581 (11280)	51.51	47.43-55.59	1507 (19673)	76.60	72.89-80.32	3467 (25412)	136.43	132.21-140.65	10425 (48061)	216.91	213.23-220.60
**Males**	**MS**	111 (625)	177.60	147.64-207.56	191 (1009)	189.30	165.12-213.47	256 (1085)	235.94	210.68-261.21	549 (2024)	271.25	251.88-290.61
	**Non-MS**	324 (6250)	51.84	46.34-57.34	794 (10004)	79.37	74.07-84.67	1488 (10765)	138.23	131.71-144.75	4300 (20085)	214.09	208.42-219.76
**41-60**													
**Overall**	**MS**	701 (2664)	263.14	246.42-279.86	1505 (5129)	293.43	280.97-305.89	2351 (7352)	319.78	309.12-330.44	3843 (13445)	285.83	278.19-293.47
	**Non-MS**	1892 (26830)	70.52	67.45-73.58	5400 (51160)	105.55	102.89-108.21	11178 (72849)	153.44	150.82-156.06	25886 (132265)	195.71	193.57-197.85
**Females**	**MS**	367 (1610)	227.95	207.46-248.44	891 (3225)	276.28	260.85-291.71	1483 (4915)	301.73	288.90-314.56	2601 (9340)	278.48	269.39-287.57
	**Non-MS**	1036 (16182)	64.02	60.25-67.79	3255 (32236)	100.97	97.68-104.26	7462 (48775)	152.99	149.79-156.18	18156 (91992)	197.36	194.79-199.94
**Males**	**MS**	334 (1054)	316.89	288.80-344.98	614 (1904)	322.48	301.48-343.47	868 (2437)	356.18	337.16-375.19	1242 (4105)	302.56	288.51-316.61
	**Non-MS**	856 (10648)	80.39	75.23-85.56	2145 (18924)	113.35	108.83-117.86	3716 (24074)	154.36	149.79-158.92	7730 (40273)	191.94	188.09-195.79
**61-80**													
**Overall**	**MS**	557 (1391)	400.43	374.68-426.18	1342 (3206)	418.59	401.51-435.67	1968 (4183)	470.48	455.35-485.60	3539 (8608)	411.13	400.73-421.52
	**Non-MS**	2880 (14581)	197.52	191.06-203.98	7688 (32506)	236.51	231.89-241.13	11420 (40963)	278.79	274.45-283.13	21552 (84384)	255.40	252.46-258.35
**Females**	**MS**	270 (776)	347.94	314.42-381.45	741 (1934)	383.14	361.48-404.81	1172 (2691)	435.53	416.79-454.26	2249 (5817)	386.63	374.11-399.14
	**Non-MS**	1389 (8139)	170.66	162.49-178.83	4188 (19684)	212.76	207.04-218.48	6890 (26609)	258.93	253.67-264.20	14364 (57512)	249.76	246.22-253.29
**Males**	**MS**	287 (615)	466.67	427.24-506.10	601 (1272)	472.48	445.05-499.92	796 (1492)	533.51	508.20-558.83	1290 (2791)	462.20	443.70-480.70
	**Non-MS**	1491 (6442)	231.45	221.15-241.75	3500 (12822)	272.97	265.26-280.68	4530 (14354)	315.59	307.99-323.19	7188 (26872)	267.49	262.20-272.78
**81-100**													
**Overall**	**MS**	72 (157)	458.60	380.65-536.54	161 (413)	389.83	342.79-436.87	302 (629)	480.13	441.08-519.17	633 (1294)	489.18	461.94-516.42
	**Non-MS**	677 (1882)	359.72	338.04-381.41	1740 (4844)	359.21	345.70-372.72	2736 (6704)	408.11	396.35-419.88	4927 (13230)	372.41	364.17-380.65
**Females**	**MS**	34 (92)	369.57	270.93-468.20	96 (271)	354.24	297.30-411.19	200 (441)	453.51	407.05-499.98	416 (926)	449.24	417.21-481.28
	**Non-MS**	353 (1120)	315.18	287.97-342.39	1029 (3128)	328.96	312.50-345.43	1874 (4893)	383.00	369.38-396.62	3382 (9621)	351.52	341.98-361.06
**Males**	**MS**	38 (65)	584.62	464.81-704.42	65 (142)	457.75	375.80-539.69	102 (188)	542.55	471.34-613.77	217 (368)	589.67	539.42-639.93
	**Non-MS**	324 (762)	425.20	390.09-460.30	711 (1716)	414.34	391.03-437.64	862 (1811)	475.98	452.98-498.98	1545 (3609)	428.10	411.95-444.24

Supplement 2 Table 6: Respiratory disease prevalence and confidence intervals over time, by sex and age-group. Abbreviations: CI=confidence interval; prev=prevalence; n=total number individuals with disease; N=total number individuals in group.

Seizures		1968-1980			1981-1990			1991-2000			2001-2012		
	Group	n (N)	Prev	CI	n (N)	Prev	CI	n (N)	Prev	CI	n (N)	Prev	CI
**6-18**													
Overall	MS	0 (67)	0	0-0	2 (72)	27.78	0-65.74	4 (95)	42.11	1.72-82.49	9 (191)	47.12	17.07-77.17
	Non-MS	0 (670)	0	0-0	1 (720)	1.39	0-4.11	5 (949)	5.27	0.66-9.87	16 (1929)	8.29	4.25-12.34
Females	MS	0 (48)	0	0-0	2 (56)	35.71	0-84.32	4 (71)	56.34	2.70-109.97	6 (136)	44.12	9.60-78.63
	Non-MS	0 (480)	0	0-0	0 (560)	0	0-0	3 (709)	4.23	0-9.01	13 (1369)	9.50	4.36-14.63
Males	MS	0 (19)	0	0-0	0 (16)	0	0-0	0 (24)	0	0-0	3 (55)	54.55	0-114.56
	Non-MS	0 (190)	0	0-0	1 (160)	6.25	0-18.46	2 (240)	8.33	0-19.83	3 (560)	5.36	0-11.40
**19-40**													
Overall	MS	18 (1753)	10.27	5.55-14.99	78 (2991)	26.08	20.37-31.79	134 (3642)	36.79	30.68-42.91	212 (6867)	30.87	26.78-34.96
	Non-MS	17 (17530)	0.97	0.51-1.43	76 (29677)	2.56	1.99-3.14	226 (36177)	6.25	5.44-7.06	655 (68146)	9.61	8.88-10.34
Females	MS	12 (1128)	10.64	4.65-16.63	48 (1982)	24.22	17.45-30.99	93 (2557)	36.37	29.11-43.63	144 (4843)	29.73	24.95-34.52
	Non-MS	13 (11280)	1.15	0.53-1.78	56 (19673)	2.85	2.10-3.59	167 (25412)	6.57	5.58-7.57	449 (48061)	9.34	8.48-10.20
Males	MS	6 (625)	9.60	1.96-17.24	30 (1009)	29.73	19.25-40.21	41 (1085)	37.79	26.44-49.13	68 (2024)	33.60	25.75-41.45
	Non-MS	4 (6250)	0.64	0.01-1.27	20 (10004)	2.00	1.12-2.87	59 (10765)	5.48	4.09-6.88	206 (20085)	10.26	8.86-11.65
**41-60**													
Overall	MS	26 (2664)	9.76	6.03-13.49	167 (5129)	32.56	27.70-37.42	386 (7352)	52.50	47.40-57.60	557 (13445)	41.43	38.06-44.80
	Non-MS	60 (26830)	2.24	1.67-2.80	355 (51160)	6.94	6.22-7.66	791 (72849)	10.86	10.11-11.61	1535 (132265)	11.61	11.03-12.18
Females	MS	13 (1610)	8.07	3.70-12.45	101 (3225)	31.32	25.31-37.33	247 (4915)	50.25	44.15-56.36	362 (9340)	38.76	34.84-42.67
	Non-MS	26 (16182)	1.61	0.99-2.22	180 (32236)	5.58	4.77-6.40	444 (48775)	9.10	8.26-9.95	969 (91992)	10.53	9.87-11.19
Males	MS	13 (1054)	12.33	5.67-19.00	66 (1904)	34.66	26.45-42.88	139 (2437)	57.04	47.83-66.25	195 (4105)	47.50	41.00-54.01
	Non-MS	34 (10648)	3.19	2.12-4.26	175 (18924)	9.25	7.88-10.61	347 (24074)	14.41	12.91-15.92	566 (40273)	14.05	12.90-15.20
**61-80**													
Overall	MS	10 (1391)	7.19	2.75-11.63	95 (3206)	29.63	23.76-35.50	227 (4183)	54.27	47.40-61.13	457 (8608)	53.09	48.35-57.83
	Non-MS	88 (14581)	6.04	4.78-7.29	403 (32506)	12.40	11.19-13.60	798 (40963)	19.48	18.14-20.82	1432 (84384)	16.97	16.10-17.84
Females	MS	8 (776)	10.31	3.20-17.42	63 (1934)	32.57	24.66-40.49	145 (2691)	53.88	45.35-62.41	301 (5817)	51.74	46.05-57.44
	Non-MS	39 (8139)	4.79	3.29-6.29	223 (19684)	11.33	9.85-12.81	468 (26609)	17.59	16.01-19.17	853 (57512)	14.83	13.84-15.82
Males	MS	2 (615)	3.25	0-7.75	32 (1272)	25.16	16.55-33.76	82 (1492)	54.96	43.40-66.52	156 (2791)	55.89	47.37-64.42
	Non-MS	49 (6442)	7.61	5.48-9.73	180 (12822)	14.04	12.00-16.07	330 (14354)	22.99	20.54-25.44	579 (26872)	21.55	19.81-23.28
**81-100**													
Overall	MS	0 (157)	0	0-0	5 (413)	12.11	1.56-22.65	22 (629)	34.98	20.62-49.33	58 (1294)	44.82	33.55-56.10
	Non-MS	0 (1882)	0	0-0	55 (4844)	11.35	8.37-14.34	158 (6704)	23.57	19.94-27.20	313 (13230)	23.66	21.07-26.25
Females	MS	0 (92)	0	0-0	5 (271)	18.45	2.43-34.47	17 (441)	38.55	20.58-56.52	41 (926)	44.28	31.03-57.53
	Non-MS	0 (1120)	0	0-0	30 (3128)	9.59	6.18-13.01	122 (4893)	24.93	20.56-29.30	198 (9621)	20.58	17.74-23.42
Males	MS	0 (65)	0	0-0	0 (142)	0	0-0	5 (188)	26.60	3.60-49.60	17 (368)	46.20	24.75-67.64
	Non-MS	0 (762)	0	0-0	25 (1716)	14.57	8.90-20.24	36 (1811)	19.88	13.45-26.31	115 (3609)	31.86	26.13-37.60

Supplement 2 Table 7: Seizure disease prevalence and confidence intervals over time, by sex and age-group. Abbreviations: CI=confidence interval; prev=prevalence; n=total number individuals with disease; N=total number individuals in group.
