# Supplementary material for: Comorbid disease burden among MS patients 1968–2012: A Swedish register–based cohort study
Source: Mult Scler. 2020 Mar 12;27(2):268–80. doi: 10.1177/1352458520910497 (PMC7820574; doi:10.1177/1352458520910497)
Supplement: MSJ910497_supplement_3 – Supplemental material for Comorbid disease burden among MS patients 1968–2012: A Swedish register–based cohort study [file MSJ910497_supplement_3.pdf]

Age 6-18 years	1968-1980 PRs	1968-1980 95% CI	1981-1990 PRs	1981-1990 95% CI	1991-2000 PRs	1991-2000 95% CI	2001-2012 PRs	2001-2012 95% CI
**Autoimmune**								
Overall	4.99	(0.46-54.31)	6.66	(1.13-39.21)	1.43	(0.18-11.50)	4.04	(1.80-9.05)
Females	5.00	(0.46-54.14)	6.66	(1.14-39.02)	2.50	(0.28-22.07)	3.59	(1.31-9.82)
Males	0	(0-0)	0	(0-0)	0	(0-0)	5.09	(1.31-19.79)
**Cardiovascular**								
Overall	0	(0-0)	0	(0-0)	0	(0-0)	10.08	(0.63-160.52)
Females	0	(0-0)	0	(0-0)	0	(0-0)	0	(0-0)
Males	0	(0-0)	0	(0-0)	0	(0-0)	0	(0-0)
**Depression**								
Overall	0	(0-0)	9.99	(0.63-158.04)	0	(0-0)	1.44	(0.62-3.34)
Females	0	(0-0)	0	(0-0)	0	(0-0)	1.22	(0.44-3.39)
Males	0	(0-0)	0	(0-0)	0	(0-0)	2.26	(0.5-10.2)
**Diabetes**								
Overall	0	(0-0)	0	(0-0)	4.99	(0.46-54.52)	3.79	(1.01-14.17)
Females	0	(0-0)	0	(0-0)	9.99	(0.63-158.01)	3.36	(0.68-16.49)
Males	0	(0-0)	0	(0-0)	0	(0-0)	5.09	(0.47-55.24)
**Renal**								
Overall	5	(1.76-14.20)	2.22	(0.77-6.38)	2.00	(0.59-6.78)	0.81	(0.19-3.39)
Females	5	(1.56-16.00)	2.67	(0.92-7.77)	2.30	(0.67-7.88)	0.96	(0.23-4.05)
Males	5	(0.48-52.62)	0	(0-0)	0	(0-0)	0	(0-0)
**Respiratory**								
Overall	0.86	(0.27-2.72)	3.40	(2.11-5.48)	1.39	(0.93-2.08)	1.18	(0.94-1.48)
Females	1.11	(0.35-3.52)	3.57	(2.12-6.01)	1.75	(1.13-2.71)	1.31	(1.01-1.70)
Males	0	(0-0)	2.73	(0.85-8.78)	0.63	(0.21-1.87)	0.90	(0.56-1.44)
**Seizures**								
Overall	0	(0-0)	19.99	(1.83-217.78)	7.99	(2.18-29.25)	5.68	(2.54-12.68)
Females	0	(0-0)	0	(0-0)	13.32	(3.04-58.34)	4.64	(1.79-12.01)
Males	0	(0-0)	0	(0-0)	0	(0-0)	10.18	(2.1-49.24)

Abbreviations: PRs=prevalence ratio, CI=confidence interval

Table 1: Prevalence ratios comparing people with MS to the general population cohort aged 6-18 years over time, and by sex per each disease category.
