# Supplementary material for: Comorbid disease burden among MS patients 1968–2012: A Swedish register–based cohort study
Source: Mult Scler. 2020 Mar 12;27(2):268–80. doi: 10.1177/1352458520910497 (PMC7820574; doi:10.1177/1352458520910497)
Supplement: MSJ910497_supplement_4 – Supplemental material for Comorbid disease burden among MS patients 1968–2012: A Swedish register–based cohort study [file MSJ910497_supplement_4.pdf]

	MS		Non-MS	
	n	%	n	%
**1968-1980**				
Type I	2	0.77	6	0.28
Type I/Type II	5	1.92	49	2.29
Type II	253	97.31	2088	97.43
**1981-1990**				
Type I	3	0.45	20	0.36
Type I/Type II	11	1.64	132	2.37
Type II	657	97.91	5428	97.28
**1991-2000**				
Type I	14	1.48	84	0.98
Type I/Type II	17	1.80	223	2.60
Type II	912	96.71	8265	96.42
**2001-2012**				
Type I	53	3.70	353	2.84
Type I/Type II	53	3.70	522	4.20
Type II	1326	92.60	11556	92.96
**2001-2012 (ICD-10)**				
Type I	151	10.54	1231	9.90
Type II	819	57.19	7410	59.61
Type Unknown	421	29.40	3549	28.55
No ICD-10 code	41	2.86	241	1.94

Abbreviations: n=total number in a specific category; ICD= International Classification of Disease

For all individuals included in a time-period (1968-1980, 1981-1990, 1991-2000, 2001-2012):

- Type I diabetes was defined as a first diagnosis < 20 years of age.
- Type II diabetes was defined as a first diagnosis > 30 years of age.
- Type I/Type II was defined as a first diagnosis between 20-30 years of age.

For individuals included under 2001-2012 ICD-10:

- Type I diabetes was defined as having an ICD-10 code (E10) without also having a type II diagnosis code at the first hospital visit.
- Type II diabetes was defined as having an ICD-10 code (E11) for type II diabetes without also having a type I diagnosis code at the first hospital visit.
- Type unknown was defined as having a diagnosis of both type I and type II diabetes at the first hospital visit (ICD-10 codes E10 or E11), or only an unspecified diagnosis of diabetes (ICD-10 codes E12-E14).
- No ICD-10 code means that the individuals was diagnosed with diabetes, but using an ICD-8/9 code in a previous time-period, with no ICD-10 code.
